# Prevalence, incidence and persistence of ADHD drug use in Japan

**DOI:** 10.1017/S2045796018000252

**Published:** 2018-05-28

**Authors:** Y. Okumura, M. Usami, T. Okada, T. Saito, H. Negoro, N. Tsujii, J. Fujita, J. Iida

**Affiliations:** 1 Research Department, Institute for Health Economics and Policy, Association for Health Economics Research and Social Insurance and Welfare, Tokyo, Japan; 2 Department of Psychiatry and Behavioral Science, Tokyo Metropolitan Institute of Medical Science, Tokyo, Japan; 3 Department of Child and Adolescent Psychiatry, Kohnodai Hospital, National Center for Global Health and Medicine, Chiba, Japan; 4 Department of Child and Adolescent Psychiatry, Nagoya University Graduate School of Medicine, Aichi, Japan; 5 Department of Child and Adolescent Psychiatry, Graduate School of Medicine, Hokkaido University, Hokkaido, Japan; 6 Graduate School of Professional Development in Education, Nara University of Education, Nara, Japan; 7 Department of Neuropsychiatry, Kindai University Faculty of Medicine, Osaka, Japan; 8 Department of Child Psychiatry, Yokohama City University Hospital, Kanagawa, Japan; 9 Department of Human Development, Faculty of Nursing, Nara Medical University, Nara, Japan

**Keywords:** ADHD, adolescent health, atomoxetine, methylphenidate, pharmacotherapy

## Introduction

There are significant geographical variations in the prevalence of attention-deficit/hyperactivity disorder (ADHD) drug use in children and adolescents (Kovess *et al.* 2015; Beau-Lejdstrom *et al.* 2016; Burcu *et al.* 2016; Piovani *et al.* 2016; Furu *et al.* 2017; Wang *et al.* 2017), although the prevalence of ADHD does not vary as a function of geographical location (Thomas *et al.* 2015). Understanding the geographical discrepancy in drug use will provide insights on potential over- or undermedication in the population. However, little is known about ADHD drug use in the Japanese population. Thus, we aimed to estimate the prevalence, incidence and persistence of ADHD drug use in children and adolescents in Japan.

## Methods

### Data source

A retrospective cohort study was conducted using the National Database of Health Insurance Claim Information and Specified Medical Checkups (NDB) that covered all electronically issued claims covered by public health insurance in Japan (Ministry of Health, Labour & Welfare, 2013; Okumura *et al.* 2017). As of April 2014, the proportion of electronically issued claims relative to all claims was 99.9% in hospitals, 95.9% in clinics and 99.9% in pharmacies. Claims for recipients of public assistance were not included in the NDB, comprising 286 048 inhabitants aged 0–19 years in 2014 (approximately 1% of the population). The NDB included information on clinical and procedural characteristics such as patient identification numbers, sex, age group, prescription date, drug codes, days of drug supply and dosage.

### Settings

Japan has 21 001 000 inhabitants aged ≤18 years (Ministry of Internal Affairs & Communications, 2015). Atomoxetine (ATX) (available since June 2009) and the osmotic-controlled release oral delivery system methylphenidate (OROS-MPH) (available since December 2007) were the only drugs approved for ADHD in the fiscal year of 2014 (between April 2014 and March 2015). OROS-MPH can be prescribed by only licensed physicians with expertise in ADHD treatment. However, immediate-release methylphenidate (IR-MPH) is not approved for ADHD treatment in Japan.

In general, the universal health insurance system of Japan pays for 70% of the medical treatment costs and the other healthcare systems (i.e. the System of Medical Payment for Services and Supports for Persons with Disabilities; and the Medical Subsidy for Children and Infants) pay for the remaining 20–30% for children and adolescents.

### Statistical analyses

We identified patients aged ≤18 years who were given at least one prescription of ADHD drug in the fiscal year of 2014. The annual prevalence of ADHD drug use was defined as the number of prevalent users per 1000 inhabitants.

Next, we identified the subgroup focusing on the incident and persistent users of ADHD drugs. First, we defined the index date as the date on which the ADHD drug was first prescribed to the patient during the fiscal year of 2014. We included the patients who had been enrolled in the database at least 180 days before and after the index date, as in previous studies (Lawson *et al.* 2012; Palli *et al.* 2012). Second, we excluded those who had a bundled payment claim within 180 days before and after the index date, in which the prescription status was not recorded. Finally, we excluded those who had a prescription of ADHD drugs within 180 days before the index date. All patients were followed up using an identification number (Kubo *et al.* 2018). The annual incidence of ADHD drug use was defined as the number of incident users per 1000 inhabitants. The percentage of persistent ADHD drug use was defined as the number of persistent users at least 150 days after the index date per 100 incident users, as in a previous study (Lawson *et al.* 2012). Discontinuation (non-persistence) was designated when a prescription medication for ADHD was not refilled within an interval defined by the days of drug supply plus a grace period of 30 days.

Subgroup analyses were conducted by sex and age groups. Age was classified into four groups: 0–6, 7–12, 13–15 and 16–18 years according to the school system (preschool, elementary school, junior high school and high school, respectively). All estimates were calculated with 95% confidence intervals (CI). All analyses were conducted using R version 3.4.1.

## Results

**Fig. 1.**
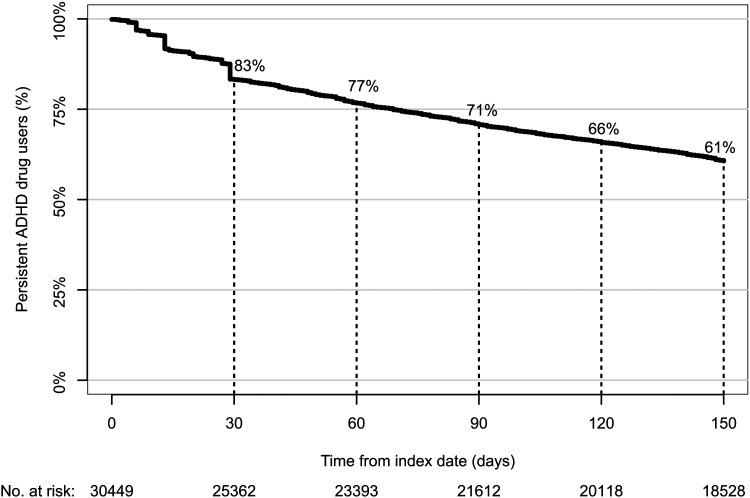
Percentage of persistent ADHD drug users.

There were 86 756 prevalent and 30 449 incident users of ADHD drugs in the database (Table 1). The annual prevalence per 1000 inhabitants was 4.1 with a peak of 7–12 years for both sexes (Table 2). Of the prevalent users, 64% used OROS-MPH (Table 1). Of the incident users, 61% still continued drug treatment at 150 days after the index date (Fig. 1). The persistence rate was much higher among those aged 7–12 years than among those aged 16–18 years (65 *v.* 43%).

**Table 1.** Sample characteristics of patients

	Prevalent user (*N* = 86 756)		Incident user (*N* = 30 449)	
Characteristic	*N*	%	*n*	%
Provider type				
Clinic	41 500	47.8	15 055	49.4
Hospital	45 256	52.2	15 394	50.6
Setting				
Inpatient	508	0.6	220	0.7
Ambulatory	86 248	99.4	30 229	99.3
Prescriber				
Non-psychiatrist	46 157	53.2	15 239	50.0
Psychiatrist	40 599	46.8	15 210	50.0
Sex				
Boys	72 520	83.6	24 528	80.6
Girls	14 236	16.4	5921	19.4
Age, years				
0–6	2659	3.1	2382	7.8
7–12	52 626	60.7	19 494	64.0
13–15	22 265	25.7	6051	19.9
16–18	9206	10.6	2522	8.3
Individual ADHD drugs				
ATX	31 491	36.3	12 876	42.3
OROS-MPH	51 013	58.8	17 384	57.1
Both	4252	4.9	189	0.6

ADHD, attention-deficit/hyperactivity disorder; ATX, atomoxetine; OROS-MPH, osmotic-controlled release oral delivery system methylphenidate.

**Table 2.** Prevalence, incidence and persistence of ADHD drug users in children and adolescents

		Prevalence		Incidence		Persistence	
Sex-age group (years)	No. of inhabitants in thousands	*n*	Annual rate per 1000 inhabitants (95% CI)	*n*	Annual rate per 1000 inhabitants (95% CI)	*n*	Percentage of 150-day persistence per 100 incident users (95% CI)
Total	21 001	86 756	4.1 (4.1–4.2)	30 449	1.4 (1.4–1.5)	18 528	60.8 (60.3–61.4)
0–6	7331	2659	0.4 (0.3–0.4)	2382	0.3 (0.3–0.3)	1523	63.9 (62.0–65.9)
7–12	6559	52 626	8.0 (8.0–8.1)	19 494	3.0 (2.9–3.0)	12 604	64.7 (64.0–65.3)
13–15	3524	22 265	6.3 (6.2–6.4)	6051	1.7 (1.7–1.8)	3322	54.9 (53.6–56.2)
16–18	3587	9206	2.6 (2.5–2.6)	2522	0.7 (0.7–0.7)	1079	42.8 (40.8–44.7)
Boys	10 755	72 520	6.7 (6.7–6.8)	24 528	2.3 (2.3–2.3)	15 282	62.3 (61.7–62.9)
0–6	3756	2262	0.6 (0.6–0.6)	2032	0.5 (0.5–0.6)	1311	64.5 (62.4–66.6)
7–12	3357	44 961	13.4 (13.3–13.5)	16 235	4.8 (4.8–4.9)	10 691	65.9 (65.1–66.6)
13–15	1806	18 459	10.2 (10.1–10.4)	4618	2.6 (2.5–2.6)	2582	55.9 (54.5–57.4)
16–18	1836	6838	3.7 (3.6–3.8)	1643	0.9 (0.9–0.9)	698	42.5 (40.1–44.9)
Girls	10 244	14 236	1.4 (1.4–1.4)	5921	0.6 (0.6–0.6)	3246	54.8 (53.5–56.1)
0–6	3574	397	0.1 (0.1–0.1)	350	0.1 (0.1–0.1)	212	60.6 (55.2–65.7)
7–12	3200	7665	2.4 (2.3–2.4)	3259	1.0 (1.0–1.1)	1913	58.7 (57.0–60.4)
13–15	1718	3806	2.2 (2.1–2.3)	1433	0.8 (0.8–0.9)	740	51.6 (49.0–54.3)
16–18	1752	2368	1.4 (1.3–1.4)	879	0.5 (0.5–0.5)	381	43.3 (40.0–46.7)

ADHD, attention-deficit/hyperactivity disorder; CI, confidence interval.

## Discussion

This is the first study to establish the representative prescribing practices of ADHD drugs in Japan. The prevalence of ADHD drug use in children and adolescents in Japan (0.4%) is much lower than that in the USA (5.3%) (Burcu *et al.* 2016) and Norway (1.4%) (Furu *et al.* 2017), while it is similar to that in Italy (0.2%) (Piovani *et al.* 2016), France (0.2%) (Kovess *et al.* 2015) and the UK (0.5%) (Beau-Lejdstrom *et al.* 2016). As in Japan, the countries with a similar prevalence have some restriction policies for prescribing ADHD drugs. In Italy, ATX and IR-MPH can be initiated only by specialists with expertise in ADHD treatment after a standardised diagnostic process (Piovani *et al.* 2016). These drugs can be re-filled by a general practitioner; however, specialists must compile an individual therapeutic plan that contains all the details on dosage and duration of therapy. In France, IR-MPH and OROS-MPH must be initiated by specialists such as child psychiatrists (Kovess *et al.* 2015). In the UK, ATX, dexamphetamine, guanfacine, lisdexamfetamine, IR-MPH and OROS-MPH are recommended to be initiated only by professionals with expertise in ADHD treatment (National Institute for Health & Care Excellence, 2006). Such a restriction policy may contribute to a relatively low prevalence of ADHD drug use in the population.

Our results indicate that children and adolescents with ADHD may be currently undermedicated in Japan. The National Institute for Health and Care Excellence guidelines recommend that pharmacological treatment should be initiated when ADHD symptoms are persistent and still causing significant impairment in at least one domain every day despite the implementation and review of environmental modifications (National Institute for Health & Care Excellence, 2018). The Regional ADHD Registry in Italy found that 44% of children and adolescents with ADHD had a severe impairment as indicated by a Clinical Global Impressions-Severity score of 5 or higher (Bonati *et al.* in press). Given the ADHD prevalence of 3.4–7.2% (Polanczyk *et al.* 2015; Thomas *et al.* 2015) and the ratio of severely impaired ADHD (Bonati *et al.* in press), 1.5–3.2% of children and adolescents would benefit from pharmacological treatment.

Among ADHD drug users, the percentage of methylphenidate use is much lower in Japan (64%) than in the UK (94%) (Beau-Lejdstrom *et al.* 2016), Norway (94%) (Furu *et al.* 2017) and Germany (75–100%) (Bachmann *et al.* 2017). This may be partly explained by the fact that the restriction policy for prescribing ADHD drugs in Japan is only applied to OROS-MPH. For instance, ATX can be prescribed by any physician in Japan. This imbalance in the restriction policy may contribute to a decrease in the use of OROS-MPH. Other explanations for the low prevalence of methylphenidate prescriptions are that IR-MPH is not approved for ADHD treatment in Japan and that both ATX and OROS-MPH are considered as the first-line drugs for ADHD treatment in the Japanese clinical practice guideline (Saito, 2016).

In addition, the persistence rate of ADHD drug use is much higher in Japan (61%) than that in the USA (10–29% at 150 days) (Lawson *et al.* 2012), while it is similar to that in the UK (66% at 1 year) according to the study conducted by Beau-Lejdstrom *et al.* who used a threefold wider grace period (Beau-Lejdstrom *et al.* 2016). We also observed a substantially lower persistence of ADHD drugs among patients who started taking drugs at an older age. These findings were consistent with those of previous studies (Beau-Lejdstrom *et al.* 2016; Wang *et al.* 2016). Future research should clarify the reason for early cessation of ADHD drugs, particularly focusing on high school grades.

The main limitation of this study is that the entire population was not accounted for in the database, which comprised 1–2% of all inhabitants. Nevertheless, our study provides representative evidence on the treatment pattern of ADHD drug use in children and adolescents in Japan.
